# New guidelines for the initial management of head injury

**DOI:** 10.1186/1741-7015-11-51

**Published:** 2013-02-25

**Authors:** Carolyn M Benson, G Bryan Young

**Affiliations:** 1 Department of Clinical Neurological Sciences, London Health Sciences Center, University Campus, 339 Windermere Road, London ON, N6A 5A5, Canada

**Keywords:** admission criteria, CT guidelines, head trauma

## Abstract

The majority of patients presenting with mild head trauma will have no intracranial pathology and can be safely discharged home. It is estimated that 10% to 15% of these patients will have clinically significant findings on computed tomography imaging and up to 1% may require neurosurgical intervention. The revised Scandinavian Head Trauma Guidelines provide an evidence- and consensus-based algorithm to assist physicians in determining which patients presenting with minimal, mild or moderate blunt head injury are at higher risk for intracranial pathology and thus require neuroimaging and hospital admission. Striking a balance between health care costs and risk of morbidity remains an ongoing challenge and we will present our concerns with this useful, but conservative management algorithm.

## Commentary

*No head injury is too trivial to be ignored*. Hippocrates

## Background

During the last 15 years, the use of computed tomography (CT) scans in mild head trauma has dramatically increased. It is estimated that 10% to 15% of this patient population will have intracranial pathology on CT and that fewer than 1% will require neurosurgical intervention. The need to identify these patients from the majority of patients that will have no intracranial pathology must be balanced against both health care costs and patient risk of exposure to ionizing radiation. Unden and colleagues [1] have conducted an extensive literature search and consensus panel to readdress the question of which patients require a CT head after minimal, mild or moderate head trauma and proposed an algorithm that will encompass most of these adult patients, including those on anticoagulation therapy. They have also provided disposition guidelines to address which patients need admission to hospital, including monitoring routines, and which can be safely discharged.

## Main text and discussion

There are many similarities between the guidelines presented by Unden and colleagues and other well-validated CT head guidelines, including the Canadian CT Head Rule and the New Orleans Criteria. Both of these established guidelines have been shown to have 100% sensitivity for the need for neurosurgical intervention and high sensitivity for detecting clinically important brain injury, ranging from 87% to 100% for the Canadian CT Head Rule and 97% to 100% for the New Orleans Criteria [2-4]. In this era of rising health care costs and finite resources, the issue has become one of specificity. These new guidelines differ in that older age, defined as greater than 60 or 65 years, as an isolated risk factor has been replaced by a combination risk factor of age greater than 65 and taking antiplatelet medication. While likely to improve the specificity of the guidelines for clinically significant intracranial pathology, it will be interesting to see the effect this will have on the sensitivity.

There has been increasing interest in biomarkers in head trauma as they are cost-effective, do not require transfer to a radiology department and do not carry the risk of radiation. These Scandinavian guidelines are the first to offer a biomarker alternative to CT scanning for low-risk patients. Unfortunately, the current lack of access to S100B testing in many centers limits the widespread use of this recommendation. In further research, it will be interesting to see whether there is good acceptance of this biomarker as a replacement for CT or whether physicians will continue to feel more comfortable with neuroimaging.

We have concerns regarding the inclusion of all shunt patients in the high-risk category. In a prospective study by Ibañez and colleagues, hydrocephalus treated with shunt insertion was found to be an independent risk factor for an acute intracranial lesion, with two out of five patients with mild head injury having positive CT scans [5]. Given the literature, altered cerebral spinal fluid dynamics, and higher prevalence of chronic subdural hematomas and hygromas in these patients, we agree that these patients should be investigated with neuroimaging after trauma. If one follows the algorithm proposed by Unden and colleagues, even after normal neuroimaging, these patients are admitted for 24 hours with close neuromonitoring. Four cases of minor head trauma resulting in shunt complications in adults have been reported and all cases have presented in a delayed fashion [6-8]. Sternbach reported the case of a 62-year-old woman who developed an acute subdural hematoma two days after mild head trauma, and her imaging suggested shunt overdrainage [7]. Gurer and colleagues reported a similar case of a 65-year-old woman who deteriorated 72 hours after a mild head injury [8]. An initial CT scan of her head was negative, but 72 hours later the CT demonstrated bilateral subdural hematomas. From the pediatric literature, a prospective observational cohort study found no difference in the incidence of clinically important traumatic brain injuries in children with and without ventricular shunts, 0.9% and 1% respectively [9]. In addition, the one child with a shunt who required neurosurgical intervention had a known chronic subdural hematoma. Blunt trauma to the shunt valve and/or tubing may result in shunt dysfunction or disconnection, but symptoms usually appear days following the incident. We acknowledge that the proportion of patients with mild head trauma and ventricular shunts will be small, and likely this recommendation will have minimal effect on health care resources, however, the effect on the individual patients should be taken into consideration. In addition, admitting these patients for 24 hours of observation may not result in earlier detection of intracranial pathology, as reported complications following mild head trauma have occurred outside this window.

Based on the Scandinavian guidelines, admission is recommended for all patients receiving anticoagulation. It is not our current practice to admit patients on anticoagulation who are neurologically intact with normal CT imaging. In a prospective study of 768 patients with a head injury who were on warfarin, four patients (0.6%) had delayed intracranial hemorrhage (days 1 to 7), resulting in two deaths [10]. If these patients were observed for 24 hours, only one of the delayed hemorrhages would have occurred during admission. A retrospective review found a similar rate of delayed hemorrhage in patients on warfarin of 1%, none of which were clinically significant [11]. Given this information, it is likely reasonable to discharge a reliable patient on anticoagulation after a normal CT scan.

## Conclusion

Unden and colleagues have provided a comprehensive management algorithm for patients with minimal, mild and moderate head injury. As the authors acknowledge, external validation of these guidelines is necessary before widespread implementation could be considered. The admission guidelines and monitoring routines suggested by the authors are very conservative and may not be feasible in some medical centers struggling with finite health care resources. Additional research is needed to determine the risk of delayed intracranial pathology in patients with risk factors such as shunt-treated hydrocephalus or anticoagulation but normal initial CT imaging.

## Abbreviations

CT: computed tomography.

## Competing interests

The authors declare that they have no competing interests.

## Authors' contributions

CB and BY drafted and revised the manuscript. Both authors have read and approved the final manuscript.

## Pre-publication history

The pre-publication history for this paper can be accessed here:

http://www.biomedcentral.com/1741-7015/11/51/prepub
